# Isotopic niche size of *Coregonus artedi* (sensu lato) Increases in the presence of *Mysis diluviana*, expanded habitat use and phenotypic diversity

**DOI:** 10.1002/ece3.6807

**Published:** 2020-09-21

**Authors:** Mark S. Ridgway, Gabriel Piette‐Lauzière, Allan H. Bell, Julie Turgeon

**Affiliations:** ^1^ Harkness Laboratory of Fisheries Research, Aquatic Research and Monitoring Section Ontario Ministry of Natural Resources and Forestry Trent University Peterborough ON Canada; ^2^ Département de biologie Université Laval Québec QC Canada

**Keywords:** cisco diversity, ecological speciation, isotopes

## Abstract

Post‐glacial colonization of lakes in Algonquin Park, Ontario, Canada resulted in food webs with cisco (*Coregonus artedi* sensu lato) and either *Mysis diluviana* or *Chaoborus* spp. as the dominant diel migrator. *Mysis* as prey, its diel movements and benthic occupancy, are hypothesized to be key elements of ecological opportunity for cisco diversity in the Laurentian Great Lakes. If correct, the hypothesis strongly implies that lakes with *Mysis* would have greater trophic niche size and drive greater adaptive radiation of cisco forms relative to lakes without *Mysis*. The dichotomy in diel migrator in Algonquin Park lakes was an opportunity to assess the isotopic niche size of cisco (δ^15^N and δ^13^C) and determine if niche size expands with *Mysis* presence. We found the presence of *Mysis* is necessary to expand isotopic niche size in our study lakes. The use of habitats not typically associated with the ancestral form of cisco (e.g., benthic habitats) and phenotypic diversity (blackfin and cisco) also continue to expand niche size in *Mysis*‐based food webs. Partial ecological speciation based on a large niche space appears to be present in one lake (Cauchon Lake) where use of alternative habitats is the only real difference in cisco. The presence of blackfin expands niche space in Cedar and Radiant Lakes. This was not matched in Hogan Lake where niche space was relatively smaller with similar forms. Possible reasons for this discrepancy may be related to the asymmetric basin of Hogan Lake and whether the two forms overlap during cool and cold‐water periods of the annual temperature cycle. By comparing trophic niche size among lakes with and without *Mysis,* we conclude that *Mysis* provides a key ecological opportunity for cisco diversity in our study lakes and likely more widely.

## Graphical abstract

*Mysis* are thought to provide an ecological opportunity in the ecology and evolution of cisco diversity. We show that in lakes with *Mysis*, cisco increase isotopic niche size and further increase niche size with expanded habitat use and phenotypic diversity. This finding has important considerations for the evolution of cisco diversity broadly.

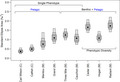

## 1 INTRODUCTION

Ecological opportunity is central to the diversifying process of adaptive radiations. Opportunity can be conceived as competitor‐free habitat and abundant resources for colonizers of islands or lakes (Losos, 2010; Martin & Wainwright, 2013; Stroud & Losos, 2016). Under these conditions, stages of radiation begin with divergence in habitat occupancy followed by trait divergence (Gavrilets & Losos, 2009; Siwertsson et al., 2013). One outcome stemming from ecological opportunity can be expanding trophic niche space allowing for diversification of an ancestral phenotype (Schluter, 1996).

Historical contingency has an important role in this process based on where early colonizers settle. Island or lake size sets upper bounds on the extent of adaptive radiations for *Anolis* sp. lizards (Losos, 1996; Losos & Ricklefs, 2009), arctic charr, *Salvelinus alpinus* (Recknagel et al., 2017), and whitefish, *Coregonus* sp. (Landry & Bernatchez, 2010; Landry et al., 2007; Praebel et al., 2013; Siwertsson et al., 2010). Adaptive radiations are sometimes replicated among islands or lakes of similar size indicating similar environmental conditions. The replicated pattern of clade diversification includes phenotypic and behavioral differences related to habitat use, replicated in similar ways among islands or lakes of similar size and environments (e.g., benthic vs. limnetic sticklebacks (*Gasterosteus*; Schluter & McPhail, 1992); European whitefish (*Coregonus lavaretus*; Ostbye et al., 2006; Lundsgaard‐Hansen et al., 2013); ciscoes (*Coregonus artedi* sensu lato) of the Laurentian Great Lakes; Eshenroder et al., 2016). Replicated radiations represent a phenotype–environment correlation between specific morphologies and niches (Harrod et al., 2010; Kahilainen et al., 2007). For *Anolis*, perching and habitat location as a function of vegetation structure represents the behavioral/habitat axis for diversification in repeated settings (Losos, 2010). For ciscoes, diel migration and foraging depth likely represent their axis for diversification repeated in several Laurentian Great Lakes (Blanke et al., 2018; Eshenroder et al., 1998; Hudson et al., 2017; Schmidt et al., 2011).

Historical contingency can also extend to the presence or absence of organisms that affect or disrupt organismal size distributions and trophic transfer. This may be particularly important in aquatic ecosystems where the size spectrum of body size and abundance are near universal features (Kerr & Dickie, 2001; Sprules & Barth, 2014). The presence or absence of keystone organisms affecting food web structure may in turn determine the presence and/or extent of adaptive radiations. Natural “experiments” in assemblage structure produced via species introductions or post‐glacial dispersal can provide insights into aquatic food web function/disruption and potentially adaptive radiations of fish.

*Mysis diluviana* (hereafter *Mysis*) is a keystone species functioning as a food web engineer capable of exerting strong influence on aquatic food web structure and function. It is a diel migrating, predatory crustacean that structures lake food webs because it governs plankton size structure as a predator, and fish productivity and trophic status as a prey (Almond et al., 1996; Nero & Sprules, 2009; Vander Zanden et al., 1999). *Mysis* introductions in lakes have disrupted food web function including plankton and planktivorous fish size structure (Devlin et al., 2017; Lasenby et al., 1986), severely reduced herbivorous cladocerans (Spencer et al., 1999), contributed to the extirpation of planktivorous fish (Lasenby et al., 1986; Spencer et al., 1991) and severed aquatic/terrestrial trophic linkages at whole lake scales (Devlin et al., 2017; Spencer et al., 1991).

*Mysis* has also been identified as a key factor in cisco diversification in the Laurentian Great Lakes (Eshenroder et al., 1998; Eshenroder & Burnham‐Curtis, 1999). Indeed, documentation of cisco form radiation in North American lakes has to date been in lakes with *Mysis* as the dominant diel migrator (e.g., Eshenroder et al., 2016; Etnier & Skelton, 2003; Piette‐Lauzière et al., 2019). Buoyancy maintenance by cisco is hypothesized as the selective mechanism for exploiting *Mysis* at depth (Eshenroder et al., 1998). The strong implication of this hypothesis is the greater trophic niche size afforded cisco in *Mysis*‐based food webs that would sustain the adaptive radiation of different cisco forms.

The distribution of *Mysis* in North American lakes is historically contingent on landscape coverage and drainage of post‐glacial lakes that formed after the last glacial maximum. Contemporary lakes with *Mysis* are relatively deep (depth > 20–30 m) and below elevations of post‐glacial lake inundation in drainage networks (Dadswell, 1974; Martin & Chapman, 1965). Above the inundation elevation, *Mysis* is absent and *Chaoborus punctipennis* (hereafter *Chaoborus*) is present as the dominant diel migrating predatory zooplankton in many lakes (Barth et al., 2014). In a recent lake survey of one such landscape, Algonquin Park in Ontario, Canada, cisco occur in lakes above and below the inundation elevation (approx. 381 m asl) of the historical drainage outflow for post‐glacial Lake Algonquin (Bell et al., 2019; Ridgway et al., 2017). Above the elevation of inundation, *Chaoborus* was present, co‐occurring cisco were monomorphic, small and occupied the pelagic habitat as would normally be associated with this species (Scott & Crossman, 1998). Below this elevation, *Mysis* was present, and cisco were either monomorphic in some lakes or showed phenotypic diversity with one form matching pelagic cisco and the other converging on a larger, *Mysis* predator with distinct black coloration on dorsal and paired fins and greater gill raker numbers (Bell et al., 2019; Piette‐Lauzière et al., 2019). In some *Mysis*‐based food webs, monomorphic ciscoes were captured in the pelagic zone as would be typical of this species while others were captured in bottom set nets that is atypical for this region. Following Eshenroder et al. (2016), the term “form” has been adopted here to describe cisco with distinct phenotypes arising from in situ ecological speciation within *C. artedi* (sensu lato) as the recognized ancestral form. Here we refer to the ancestral, small pelagic form as cisco and the larger *Mysis* predator that has evolved in several lakes as blackfin (Bell et al., 2019; Piette‐Lauzière et al., 2019).

Diel movements and size/phenotypes of cisco (sensu lato) show diverse patterns whether *Chaoborus* or *Mysis* are the dominant diel migrators. In *Chaoborus*‐based food webs, cisco size is related to zooplankton density with complex diel patterns of movement including no diel movement (Ahrenstorff et al., 2013). In non‐*Mysis* food webs in a Minnesota lake region (*Chaoborus* may or may not be present), size and shape of cisco are related to overall lake productivity with use of shallow habitat occurring in lakes with inadequate hypolimnetic oxythermal habitat (Jacobson et al., in review). In response, cisco fin shape and size shifted to accommodate a more substrate‐based food source. The typical small pelagic form of cisco was found in oligotrophic lakes with enough hypolimnetic dissolved oxygen.

In *Mysis*‐based food webs, such as the Laurentian Great Lakes, cisco have radiated into different forms along a depth gradient (Eshenroder & Burnham‐Curtis, 1999; Eshenroder et al., 2016). Exploiting *Mysis* during diel movement or during benthic stages is an important element of Laurentian Great Lake cisco diversity (Ahrenstorff et al., 2011; Hrabik et al., 2006; Jensen et al., 2006; Sierszen et al., 2014). Determining the mechanism(s) governing trade‐offs in diel movement and preferred resting depths for cisco in any lake food web are complex and establishing the relative importance of factors such as minimizing predation, maximizing growth potential, or optimizing oxythermal habitat selection all have different levels of support in different lake ecosystems (Ahrenstorff et al., 2013; Hrabik et al., 2006; Jensen et al., 2006). While advances are being made in understanding cisco diversity (e.g., Ahrenstorff et al., 2013; Piette‐Lauzière et al., 2019; Turgeon et al., 1999, 2016), insight into how food web structure fundamentally drives cisco diversity remains an elusive topic.

In this study, we return to the early observation of *Mysis* in diets of different cisco forms as originally described by Koelz (1929) and the hypothesis that *Mysis* is important for cisco diversity (Eshenroder et al., 1998). The historical contingency of *Mysis* presence/absence in lake food webs in Algonquin Park and cisco distribution in the region allows for a comparison of cisco trophic ecology between *Mysis* and *Chaoborus* lake food webs. We compare cisco in *Mysis* versus *Chaoborus* food webs using isotopic trophic niche size (Jackson et al., 2011; Swanson et al., 2015) and pose several hypotheses. Because cisco also occupy different habitats and/or have different phenotypic forms in Algonquin Park lakes with *Mysis* (Bell et al., 2019; Piette‐Lauzière et al., 2019), we hypothesize that cisco (sensu lato) in these lake food webs will show greater isotopic niche size corresponding to greater habitat and/or phenotypic diversity (Eshenroderet al., 1998; Eshenroder & Burnham‐Curtis, 1999), relative to *Chaoborus*‐based food webs. Finally, we expect to find differences in the trophic niches of sympatric forms or fish found in pelagic versus benthic habitats within *Mysis* lakes. Given the evidence uncovered in this study, we expand on the *Mysis*/depth hypothesis for cisco diversity.

## 2 METHODS AND MATERIALS

### 2.1 Study Lakes

**FIGURE 1:**
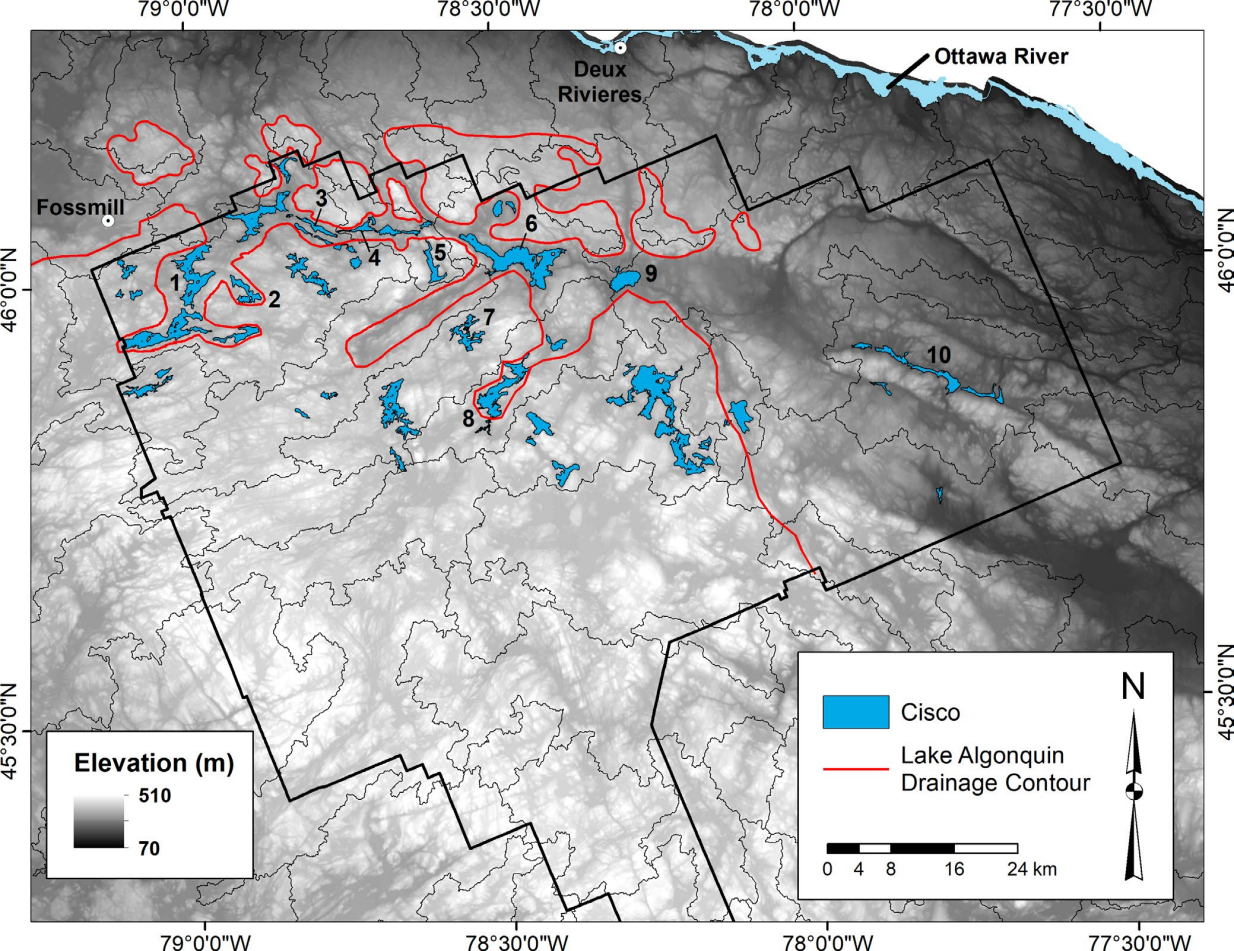
The native distribution of cisco (*Coregonus artedi* sensu lato) in a digital elevation map of Algonquin Park. Light areas are relatively high elevation and dark areas are lower elevation. The park boundary is in black (solid line). The extent of *Mysis diluviana* presence is defined by the red line matching an elevation of 381 m. The lower elevation area bounded by the 381 m contour is the area of inundation by Lake Algonquin drainage beginning in the west at the town of Fossmill and flowing east. Cisco colonized lakes within the drainage system and lakes above the 381 m contour. Study lakes are numbered as: (1) Manitou; (2) Three Mile; (3) Mink; (4) Cauchon; (5) Carl Wilson; (6) Cedar; (7) Catfish; (8) Hogan; (9) Radiant; (10) Grand

Figure 1 shows the native distribution of cisco on the Algonquin Park landscape based on recent and historical lake surveys (Bell et al., 2019). Cisco occupy lakes within the valley system that drained pro‐glacial Lake Algonquin from the Fossmill Outlet in the west to the east (approx. 13,000–12,000 cal yrs BP) as represented by the lower elevation (dark landscape on the gray scale in Figure 1) (Dyke, 2004; Harrison, 1972; Karrow, 2004; Lewis et al., 1994). Cisco above this elevation are native populations (no stocking history) and presumably arrived via upstream movements during Lake Algonquin drainage. *Mysis* occupancy in lakes below 381 m asl (red dotted line in Figure 1) and absence above this elevation is the “fingerprint” of Lake Algonquin inundation in the valley (Martin & Chapman, 1965; Dadswell, 1974). Because cisco distribution is centered on the Lake Algonquin drainage system, we presume this defines the entry point of cisco to the landscape via the Fossmill Outlet (Mandrak & Crossman, 1992). Lakes used in this study are numbered in Figure 1 and their characteristics are listed in Table 1.

**TABLE 1:** Location and characteristics of the cisco study lakes in Algonquin Provincial Park

Lake name	Latitude	Longitude	Surface area (ha)	Mean depth (m)	Max. depth (m)	Volume (x10^6^ m^3^)
Carl Wilson	46.015	−78.602	374.2	9.8	23.4	36.3
Catfish	45.938	−78.549	528.9	6.7	22.7	8.8
Cauchon	46.062	−78.714	232.2	13.5	43.0	30.8
Cedar	46.020	−78.464	2,529.2	13.5	58.5	340.0
Grand	45.876	−77.805	169.6	8.6	42.3	64.6
Hogan	45.873	−78.497	1,283.5	7.4	38.2	89.7
Manitou	46.015	−78.992	1,381.9	13.6	38.4	187.1
Mink	46.063	−78.789	227.9	15.6	45.7	35.1
Radiant	45.993	−78.291	638.1	8.8	36.5	55.8
Three Mile	45.992	−78.909	415.0	11.3	41.3	46.4

Carl Wilson and Catfish lakes, both with *Chaoborus*, are situated in different watersheds and are directly connected by drainage to the lower elevation lakes (#5 and #7; Figure 1). All other lakes are in the lower elevation region. Manitou and Three Mile lakes have been separated from the other lakes in this drainage system for at least 10,000 cal yrs BP (Harrison, 1972; Ridgway et al., 2017). Similarly, the Mink Lake Sill between Mink Lake (#3; Figure 1) and Cauchon Lake (#4; Figure 1) has separated their respective watersheds for approximately 12,000 cal yrs BP. Hogan Lake (#8; Figure 1) has been separate from all other lakes for a similar length of time (Ridgway et al., 2017). All other lakes drain eastward in the Petawawa R drainage with a series of waterfalls preventing any upstream movement beginning with Grand Lake (#10; Figure 1).

### 2.2 Netting surveys

Netting surveys to acquire isotope samples followed the same protocol as described in Bell et al. (2019). Briefly, bottom set gillnets were used as part of the park‐wide survey of fish in lakes. Bottom net surveys followed a depth stratified random design allocating netting effort in proportion to the aerial surface area of depth strata (1–3 m, 3–6 m, 6–12 m, 12‐20m, 20–30 m, 30+ m). Nets (25 m long × 2 m high; 64 m long × 2 m high) were set for 1 or 2 hr and consisted of a mesh series ranging from 38 to 127 mm mesh panels (Bell et al., 2019). Some lakes were surveyed for ciscoes using nets with 38, 51, and 64 mm mesh only to increase sample size. Pelagic nets of similar mesh series were set in mid‐lake over a deep basin in early evening hours in all study lakes. Fish were removed from the nets and placed on ice for processing on shore. Tissue samples were stored on ice in the field and later frozen.

### 2.3 Tissue sampling and processing

All samples were obtained between 2010 and 2016 by members of the Harkness Laboratory of Fisheries Research. A sample of back muscle without skin or scales was taken from fish (see Table 2 for sample sizes). Mussels (*Elliptio complanata*) (*N* = 8–19) were sampled from each lake in shallow water (≈1 m) during the week of netting. Mussels were sampled to acquire baseline estimates of carbon (δ^13^C) and nitrogen (δ^15^N). Each sample was dried at 60°C for 48 hr, ground into a fine powder, and stored in glass vials. Samples were sent to the Stable Isotopes in Nature Laboratory (SINLAB) at the University of New Brunswick for stable isotope analysis.

**TABLE 2:** Sample sizes for cisco isotope estimation for each study lake

Lake	Cisco samples
Carl Wilson	22
Catfish	30
Cauchon	29
Cedar	92
Grand	30
Hogan	57
Manitou	15
Radiant	77
Three Mile	68

### 2.4 Stable isotopes measurements

Measurements of stable isotopes were reported in parts per thousand (‰) relative to the international standards (Vienna Pee Dee Belemnite for carbon and atmospheric air for nitrogen). Isotopes values were also normalized using secondary standards (see SINLAB procedures) to allow comparisons over multiple years.

A carbon and nitrogen ratio (C:N) between 3.5 and 4.5 (this study: 3.9) can indicate interference of lipids in samples. Several arithmetic corrections have been suggested to correct the δ^13^C values based on the correlation of lipid content and the C:N ratio (Kiljunen et al., 2006; Logan et al., 2008; McConnaughey & McRoy, 1979). A previous study on ciscoes (Schmidt et al., 2011) applied the Kiljunen et al. (2006) equation to control for lipid bias in their fresh samples. We did the same with our samples.

### 2.5 Baseline correction

Lakes differ in their δ^13^C and δ^15^N at the base of their food chain (e.g., mussel tissue profiles). Thus, before attempting among lake comparisons, corrections of these baselines must be performed. Cabana and Rasmussen (1996) recommended unionid mussels (such as *E. complanata*) for the baseline correction. *E. complanata* has a slow nitrogen turnover due to their long life and relatively large size. We used bi‐plots of mussel δ^13^C and δ^15^N by lake to determine centroid points as baselines (Table 3). The lake‐centroid δ^13^C and δ^15^N from mussels was subtracted from individual cisco δ^13^C and δ^15^N signatures to obtain relative isotopic values for the consumers (i.e., ciscoes).

**TABLE 3:** Baseline centroid values for δ^15^N and δ^13^C based on samples of *Elliptio complanata* in the study lakes

Lake name	*N*	δ^15^N	δ^13^C
Carl Wilson	10	3.8149	−26.4991
Catfish	11	4.1639	−29.2437
Cauchon	10	2.4920	−27.4665
Cedar	10	3.2930	−28.3426
Grand	19	2.7877	−26.6745
Hogan	10	2.7657	−27.5857
Manitou	9	2.3205	−25.2864
Mink	8	2.5414	−27.8741
Radiant	17	3.3578	−27.9997
Three Mile	9	2.8422	−24.5808

### 2.6 Analysis

Isotopic niche size of cisco was compared among lakes differing in dominant diel migrator (*Mysis* vs. *Chaoborus*), habitat of capture location (pelagic vs. benthic nets), and phenotypic diversity (lakes monomorphic form of *C. artedi* vs. lakes with blackfin and cisco forms). Each category (diel migrator, habitat, and phenotype) potentially represents escalating levels of niche expansion. Four lakes had monomorphic cisco captured only in pelagic nets; of these, two lakes had *Chaoborus* (Carl Wilson and Catfish Lakes) and two lakes had *Mysis* (Manitou and Grand) as the dominant diel migrators. Five lakes had pelagic and benthic captures of cisco (Three Mile, Cauchon, Cedar, Hogan, and Radiant Lakes) with three lakes in this set also having phenotypic diversity as represented by the presence of blackfin and cisco forms (Cedar, Hogan and Radiant Lakes) (Bell et al., 2019; Piette‐Lauzière et al., 2019).

We estimated niche size using the Bayesian standard ellipse area (SEA_B_) of SIBER (R package SIBER; Jackson et al., 2011). The SEA_B_ is based on the covariance matrix between isotopes (in our case δ^13^C and δ^15^N) using the posterior estimate of the standard conjugate prior of a covariance matrix simulated by MCMC (Jackson et al., 2011). Draws from the MCMC provide estimates of the length (*x* axis) and width (*y* axis) of ellipses and the associated covariance matrixes. We used default settings for the MCMC (vague priors; 1,000 iteration burn in; 2 chains of 20,000 iterations thinned by 90% providing a final 4,000 iterations for posterior estimates) (Jackson et al., 2011). Box plots of posterior estimates for SEA_B_ are presented with the median, 50% credible intervals (smallest box), 75% credible intervals, and 95% credible intervals, CI; (largest box).

We first combine all cisco in a lake to represent the overall *C. artedi* (sensu lato) isotopic niche, regardless of capture location or phenotypic form. This species is widely recognized as diverse and deserving of the taxonomic qualifier “sensu lato.” Therefore, combining all cisco allowed for among lake comparisons. Our intention was to describe the *C. artedi* niche space broadly defined for each lake to assess whether niche space changed across the lake set. We interpreted non‐overlap of the 95% credible intervals as a significant or strong difference when comparing isotopic niche size among lakes or between lakes with *Chaoborus* versus lakes with *Mysis*.

Second, we estimated isotopic niche overlap between ciscoes captured in benthic or pelagic nets (sample sizes in Table 4). This overlap was expressed as a probability using nicheROVER (R package; Swanson et al., 2015). We choose nicheROVER because of the advantages of probability comparison (and 95% credible intervals) over geometric or space comparisons (Swanson et al., 2015). Overlap comparisons (mean, 95% credible intervals) are summarized as, for example, cisco captured in benthic habitat overlapping with pelagic captured cisco from the same lake. Similarly, cisco captured in pelagic habitat overlapping with cisco captured in benthic habitat. nicheROVER allows for asymmetric comparisons of this kind which is useful in assessing potential directionality in how niche space may have shifted in two‐way comparisons.

**TABLE 4:** Total sample size per lake for cisco Isotope estimation for benthic and pelagic captures

Lake	Gear	Count by gear
Three Mile	Benthic	34
Three Mile	Pelagic	34
Cauchon	Benthic	8
Cauchon	Pelagic	21
Cedar	Benthic	59
Cedar	Pelagic	33
Hogan	Benthic	30
Hogan	Pelagic	27

Third, for lakes with both pelagic and benthic captures of ciscoes (sample sizes in Table 5), we used principle component analysis (PCA) to examine the association between phenotype (length, shape, total gill raker count; Piette‐Lauzière et al., 2019), isotopic niche (δ^13^C and δ^15^N), and environment (pelagic versus. benthic). Data from geometric morphology procedures incorporating 22 homologous landmarks (figure 2 in Piette‐Lauzière et al., 2019), measurements, and gill raker counts were drawn from Piette‐Lauzière et al. (2019), including whether individual cisco were captured in pelagic or benthic nets. Landmarks were used to adjust for size and incorporate shape. Briefly, size effects were removed using ordinary least square residuals from all linear measurement's versus standard length for all lakes combined (Reist, 1985). Standard length was based on landmarks 1 and 2 in figure 2 of Piette‐Lauzière et al. (2019). Accounting for size in this manner was consistent with earlier publications on cisco morphology (Muir et al., 2013; Reist, 1985). Briefly, shape analysis was based on Procrustes superimposition initially by alignment on landmarks 1 and 2 on each fish sample and then superimposed on their centroids. Procrustes coordinates and shape variation generated a covariance matrix that was then used in the PCA analysis as shape (Piette‐Lauzière et al., 2019).

**TABLE 5:** Total sample size per lake for cisco forms in Cedar and Hogan Lakes

Lake	Raker form	Count by raker form
Cedar	High	44
Cedar	Low	25
Hogan	High	14
Hogan	Low	37

Note

Gill raker form defined as the low raker form (gill rakers counts ranging from 40 to 51) and high raker form (gill rakers counts = 52+) based on Bell et al. (2019). Not all benthic or pelagic net samples identified in Table 3 were counted for gill rakers.

Because a depth gradient is hypothesized to be the axis of ecological opportunity for cisco, the ordination analysis provided a comparison between the niche of the ancestral state form of cisco (small pelagic form) and that of the benthic form. A total of 151 individuals caught in benthic (BEN) or pelagic (PEL) habitat from four lakes (Cedar, Hogan, Cauchon, and Radiant) were characterized for all these variables. Mink Lake had no captures of pelagic cisco and typical cisco was absent. Radiant Lake had relatively few pelagic captures of cisco (*N* = 7) but was included to assess the predominantly benthic blackfin in Radiant Lake with respect to ordinations of lakes with pelagic and benthic captures of fish (Cedar, Hogan and Cauchon). Piette‐Lauzière et al. (2019) identified HGR/LGR populations in Cedar and Hogan Lakes as the most differentiated groups.

We determined the number of components to retain from the PCA by the broken stick model (Peres‐Neto et al., 2003). This statistical procedure simulates randomly broken *K* pieces (*K* = number of variables) from a stick. Pieces sorted in descending order of proportion represent the threshold level of the random distribution of explained variance. Significant PC components were analyzed by ANOVA with habitat (benthic or pelagic capture) and lake (Cedar, Hogan, Radiant, and Cauchon Lks) as factors to assess the relative contribution of each to the multivariate patterns exposed by the PCA. Based on the ANOVA, within lake post hoc comparisons were performed using Tukey HSD between pelagic and benthic‐captured cisco. Post hoc comparisons among lake were also conducted to assess any differences that may exist at the lake level.

Finally, given the results of the PCA and the potential association between phenotype and environment among lakes, we further explored differences in isotopic niche size by comparing blackfin and cisco forms residing in Cedar and Hogan Lakes (Bell et al., 2019; Piette‐Lauzière et al., 2019). We were particularly interested in the association between δ^13^C and δ^15^N and the bimodal gill raker counts in both lakes. NicheROVER was used to compare the probability of overlap between to the two forms. Baseline data were used to estimate trophic position of cisco and blackfin in Cedar and Hogan Lakes following Vander Zanden et al. (1999). Centroid values for δ^15^N for *Eliptio complanata* (trophic level 2) were used as the baseline estimate for each lake (Table 3). Trophic position for cisco and blackfin was calculated as:

$$\text{Consumer Trophic Position}\;\left(\text{TP}\right)=\left(\delta^{15}N\;\text{consumer}‐\delta^{15}N\;\text{baseline}\right)/3.4+2$$

where 3.4 is the per trophic level increase in $\delta^{15}N$ $\left(o\;/\text{oo}\right)$. Wilcoxon rank sum test was used to determine if TP for blackfin was significantly greater than TP for cisco because TP data did not satisfy the normality assumption.

## 3 RESULTS

**FIGURE 2:**
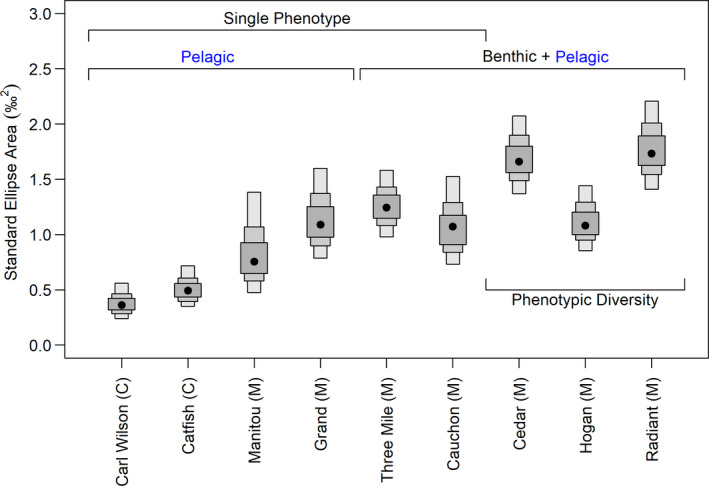
The Standard Elipse Area (SEA_B_; o/oo^2^) of cisco (*C. artedi* sensu lato) for the lake set. The dominant diel migrator is either *Chaoborus* (=C) or *Mysis* (=M) among the lake set. Single phenotype refers to cisco that are the typical form for the species including pelagic only habitat occupancy, gill raker distribution, and size. Pelagic and benthic habitat refers to cisco captured in bottom set gillnets and pelagic gillnets. Phenotypic diversity refers to either two forms (blackfin and cisco forms; Cedar and Hogan Lakes) or blackfin form primarily with relatively few cisco forms (Radiant Lake). Box plots of posterior estimates for SEA_B_ are presented with the median (point), 50% credible intervals (smallest box), 75% credible intervals, and 95% credible intervals (largest box)

Isotopic niche space for cisco differed across lakes in Algonquin Provincial Park (Figure 2). Generally, all lakes with *Mysis* as the dominant diel migrator had greater niche space than lakes with *Chaoborus* as the dominant diel migrator regardless of habitat use and the extent of phenotypic diversity. Only Manitou Lake overlapped slightly (based on lower 95% CI) with both *Chaoborus* lakes (Carl Wilson and Catfish Lakes). The standard ellipse area (SEA_B_) for lakes with *Chaoborus* revealed relatively small and more restricted isotopic niche sizes (Figure 2; Carl Wilson, med. SEA_B_ = 0.38, 95% Credible Interval 0.24, 0.56; Catfish, med. SEA_B_ = 0.51, 95% CI 0.35, 0.72).

Manitou and Grand Lakes, both with *Mysis* and monomorphic cisco, had greater isotopic niche space relative to *Chaoborus* lakes by a factor of 1.5–3× based on ratios of median SEA_B_. Because the lower 95% CI of Manitou Lake overlapped with both Carl Wilson and Catfish Lakes (Figure 2; Manitou, med. SEA_B_ = 0.85, 95% CI 0.48, 1.38), the greater isotopic niche space of Manitou Lake was not a strong difference relative to lakes with *Chaoborus*. The lower 95% CI for Grand Lake exceeded the upper 95% CI for both *Chaoborus* lakes indicating a strong difference in isotopic niche size between those lakes and the greater isotopic niche size in Grand Lake (Figure 2; med. SEA_B_ = 1.15, 95% CI 0.79, 1.60).

The pattern of increased isotopic niche size for the remaining *Mysis* lakes continued relative to *Chaoborus* lakes. Three Mile Lake (med. SEA_B_ = 1.26, 95% CI 0.98, 1.58) and Cauchon Lake (med. SEA_B_ = 1.08, 95% CI 0.73, 1.53) also have monomorphic cisco populations but differed from Manitou and Grand Lakes because cisco occupancy included both pelagic and benthic habitats based on net captures (Figure 2). Both lakes had non‐overlapping 95% CI relative to the *Chaoborus* lakes indicating a greater isotopic niche size relative to those lakes. The greater occupancy by cisco of the two general lake habitats in Three Mile and Cauchon Lakes did not clearly increase the SEA_B_ of each population relative to Manitou and Grand Lakes based on overlapping 95% CI. Three Mile Lake cisco appear to have a slightly greater niche space than the other monomorphic populations in *Mysis*‐based food webs.

Generally, there were clear, strong differences in isotopic niche size between the two *Chaoborus* lakes versus the three lakes with phenotypic diversity (Figure 2). In two of the three lakes with blackfin and cisco (Cedar and Radiant), SEA_B_ was larger than all other lakes with little overlap in 95% CI with other lakes (Figure 2). The overall niche space of cisco in Cedar Lake (med. SEA_B_ = 1.70, 95% CI 1.37, 2.07) and Radiant Lake (med. SEA_B_ = 1.78, 95% CI 1.41, 2.21) was 3–4× greater (ratio of median SEA_B_) than in lakes with *Chaoborus*. The lower 95% CI of Cedar Lake SEA_B_ barely overlapped with the upper 95% CI of Manitou Lake SEA_B_.

The exception to increases in isotopic niche space among lakes with phenotypic diversity is Hogan Lake (Figure 2). It contains both blackfin and cisco forms with fish captured in both pelagic and benthic deployed nets. The isotopic niche space (med. SEA_B_ = 1.13, 95% CI 0.85, 1.44) was equivalent to other lakes with monomorphic cisco but smaller than the other phenotypically diverse populations.

**FIGURE 3:**
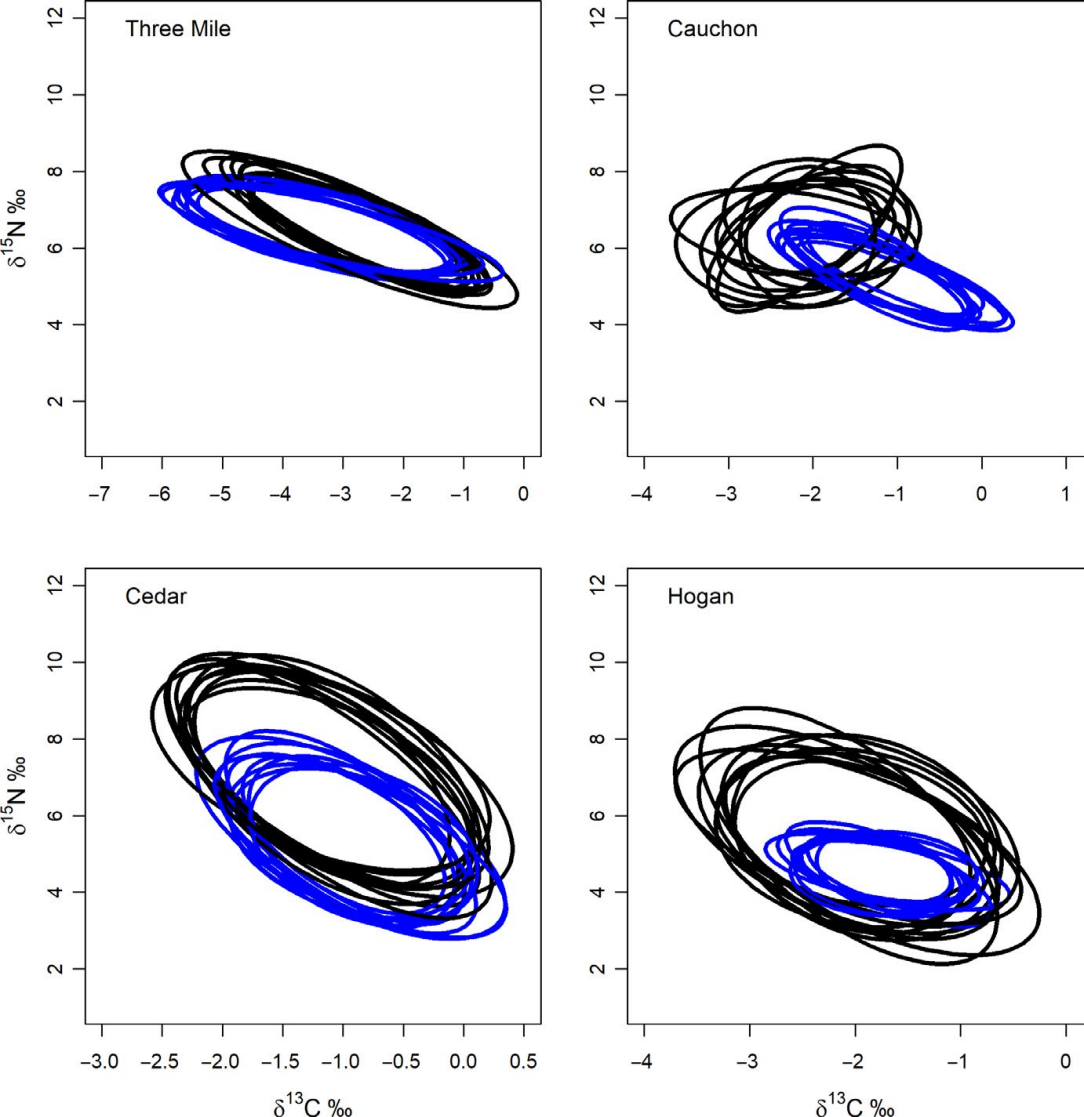
Plausible isotopic niche space for ciscoes in lakes with both pelagic (blue) and benthic (black) captures of ciscoes. Axis values are δ^15^N and δ^13^C relative to baseline values for each lake (δ^15^N_fish_ − δ^15^N_base_; δ^13^C_fish_ − δ^13^C_base_). Note the scale of relative δ^13^C varies with each figure panel

Four of the *Mysis*‐based cisco populations had enough catches in both pelagic and benthic nets to estimate niche size for fish captured in each of the two habitats (Figure 3). Two lakes with blackfin (Mink and Radiant) did not have enough pelagic catches to make a comparison of niche size between fish in the two habitats. Among the four *Mysis* lakes, isotopic niche size in general appeared to vary more on the δ^13^C axis than on the δ^15^N axis overall (Figure 3). All four lakes showed overlap in isotopic niche size with the greatest overlap occurring in Three Mile Lake (Table 6; Figure 3). Both pelagic‐ and benthic‐captured cisco in Three Mile also had similar sized isotopic niches (1.22 and 1.08 SEA_B_, respectively; Table. 3) accompanying high levels of overlap (Table 6; Figure 3). Although Cauchon Lake is regarded as having a single phenotype of cisco, the probability of overlap for fish captured in benthic and pelagic nets was lowest among the lake set (prob. of overlap with benthic = 33.85; with pelagic = 36.45; Table 6; Figure 3). For both Three Mile and Cauchon Lakes, there was no clear indication of directionality in overlap between cisco captured in different habitats although they strongly differed in the magnitude of overlap (Table 6).

**TABLE 6:** Summary of isotopic niche size as standard ellipse area (SEA_B_) and probability of overlap for ciscoes captured in pelagic and benthic nets (see Figure 3)

Lake	Capture habitat	SEA_B_ median	95% CI	Overlap with	Prob. of overlap	95% CI
Three Mile	Benthic	1.08	0.75–1.51	Pelagic	84.9	65.0–94.4
Three Mile	Pelagic	1.22	0.85–1.68	Benthic	70.5	54.2–83.1
Cauchon	Benthic	1.11	0.45–2.13	Pelagic	33.8	12.3–67.0
Cauchon	Pelagic	0.53	0.33–0.80	Benthic	36.5	8.5–74.5
Cedar	Benthic	1.51	1.16–1.94	Pelagic	48.9	35.6–65.9
Cedar	Pelagic	1.06	0.74–1.46	Benthic	81.4	65.7–92.2
Hogan	Benthic	1.56	1.05–2.20	Pelagic	41.4	28.2–55.9
Hogan	Pelagic	0.42	0.28–0.60	Benthic	97.2	85.6–100.0

Note

Isotopic niche space was estimated from SIBER and probability of overlap from nicheROVER. 95% CI refers to 95% credible intervals.

There is directionality in niche space between benthic and pelagic cisco in Cedar and Hogan Lakes with pelagic cisco niche space being smaller and largely contained within the larger niche space of benthic‐captured cisco (Table 6). Hogan Lake had the greatest difference between fish occupying the two habitats (1.56 SEA_B_ benthic captures; 0.42 SEA_B_ for pelagic captures). There was evidence of directionality in niche overlap between benthic and pelagic captured cisco in both lakes. The probability of overlap by benthic cisco with pelagic cisco in both lakes was less than 50% (Cedar Lake prob of overlap = 48.9; Hogan Lake prob. = 41.4; Table 6), with 95% credible intervals below overlap levels of pelagic captured cisco with benthic‐captured cisco. In contrast, the probability of overlap of pelagic cisco with benthic cisco was clearly greater in both lakes (prob. of overlap of pelagic cisco with Cedar benthic captures = 81.38; with Hogan benthic captures = 97.24; Table 6).

We examined PCA ordination patterns among the lake set in Figure 3 except for Three Mile Lake which showed extensive niche overlap (Table 6; Figure 3). We added Radiant Lake benthic captures of blackfin to the analysis to assess their position relative to lakes with blackfin and cisco forms (Cedar and Hogan Lakes).

**FIGURE 4:**
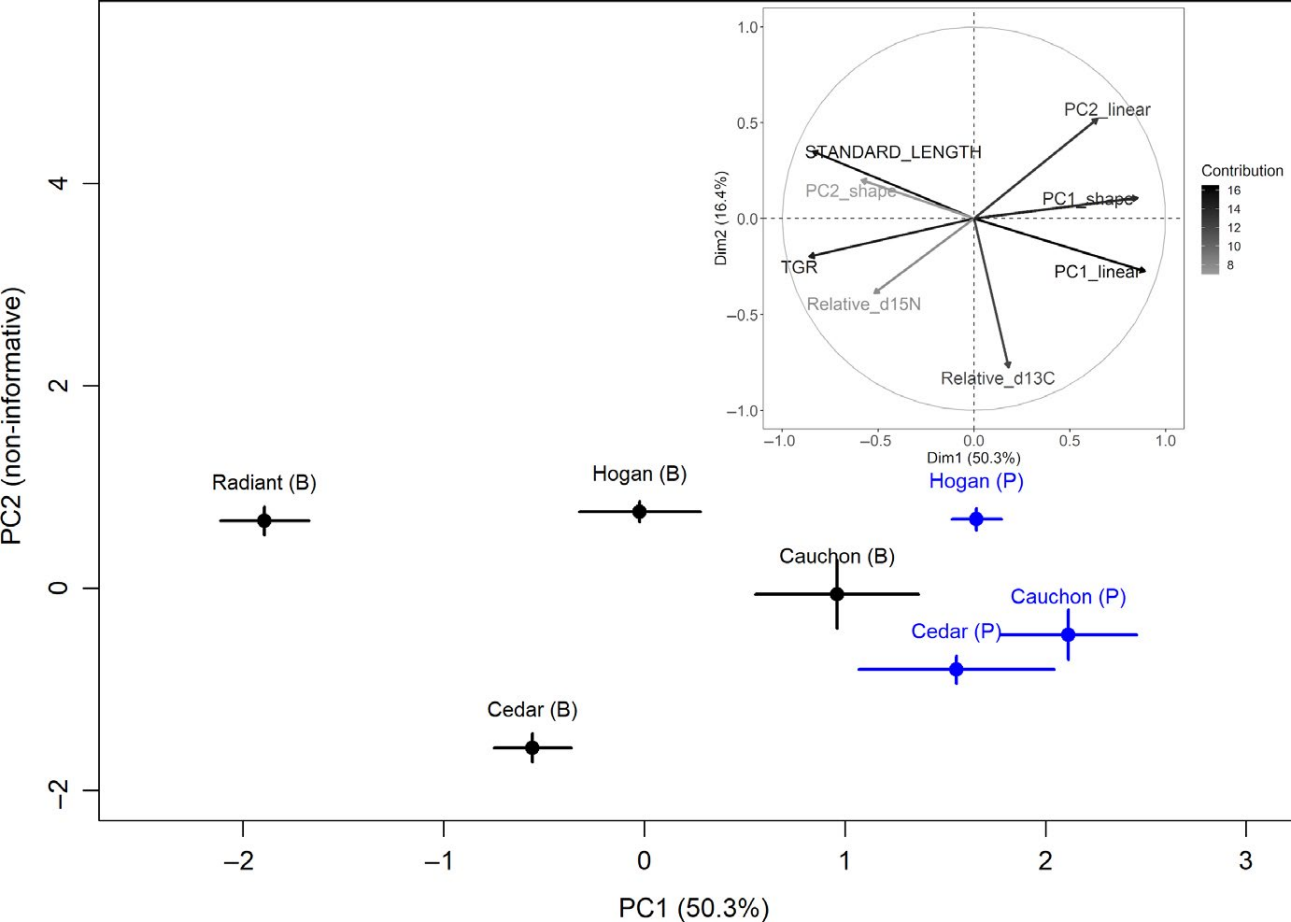
PCA analysis of ciscoes in lakes where pelagic and benthic nets captured ciscoes. Phenotypic (size, shape, and gill raker number) and isotopic niche data are included in the PCA where all variables were recorded for each specimen. Morphological data based on digitized landmarks from Piette‐Lauzière et al. (2019). Insert is the pattern of variable loading of PC1 and 2 in the PCA and associated gray scale is the percent of variation accounted for in the loadings

The first two components of the PCA accounted for 66.6% of variation, but only PC1 exceeded the threshold level determined by the broken stick model (50.3% vs. 16.3% of variation explained) (Figure 4). The ANOVA with habitat and lake identity as factors was based on PC1. Both habitat and lake effects were significant with habitat accounting for greater variation (*F* = 99.55, *df* = 1; *p* < .001) than lake identity (*F* = 12.75, *df* = 4; *p* < .001). The interaction between habitat and lake identity was not significant (*F* = 0.62, *df* = 2; *p* = .497). Most of the variation in lake identity was attributable to the effects of Radiant Lake relative to the rest of the lake set (Tukey's HSD with other lakes; diff. from Cauchon = −2.01, *p* < .001; diff. from Cedar = −1.15, *p* = .002; diff. from Hogan = −1.54, *p* < .001).

Variables previously identified as contributing to phenotypic diversity in Algonquin Park cisco populations were important in explaining the pattern of phenotype and environment correlation in PC1 (Figure 4). Loadings of eigenvectors pointed to PC1 of linear size traits, and total gill rakers as important (Figure 4 insert), and PC1 and PC2 of shape as strong contributors to ordination patterns (Figure 4; insert). Relative δ^15^N also contributed to PC1 but was relatively weaker than the size, shape, and habitat elements in explaining patterns. Relative δ^15^N was also less influential than relative δ^13^C in influencing ordination patterns. The relative proximity in PC ordination space of all pelagic cisco samples for Cedar, Hogan, and Cauchon Lakes points to a well‐defined morphological grouping combined with a relatively small niche space (small apparent effect of δ^15^N) for this group of cisco (Figure 4). Based on Tukey's HSD, the pelagic cisco in each of the three lakes did not differ from each other in PC1 (HSD; all two‐way comparisons among Cedar, Hogan and Cauchon Lakes, NS). Based on PC1, benthic‐captured cisco and blackfin in the lake set occupied greater ordination space than pelagic captured cisco.

Interestingly, benthic and pelagic captured cisco in Cauchon Lake indicated some separation in ordination space but the difference was not significant (Figure 4; Tukey HSD; diff. between Cauchon pelagic and benthic cisco = 1.154, *p* = .862), and not enough for different forms to be recognized in the field.

The ANOVA revealed habitat (benthic vs. pelagic captures) as the strongest effect in explaining the patterns in PC1 in general. For the two lakes with cisco and blackfin forms (Cedar and Hogan Lakes; Figure 4), comparisons showed that benthic/pelagic differentiation observed on PC1 was significant in Cedar (Tukey HSD; diff. between pelagic and benthic samples = 2.112, *p* < .001) and Hogan Lakes (Tukey HSD; diff. between pelagic and benthic samples = 1.678, *p* = .002).

**FIGURE 5:**
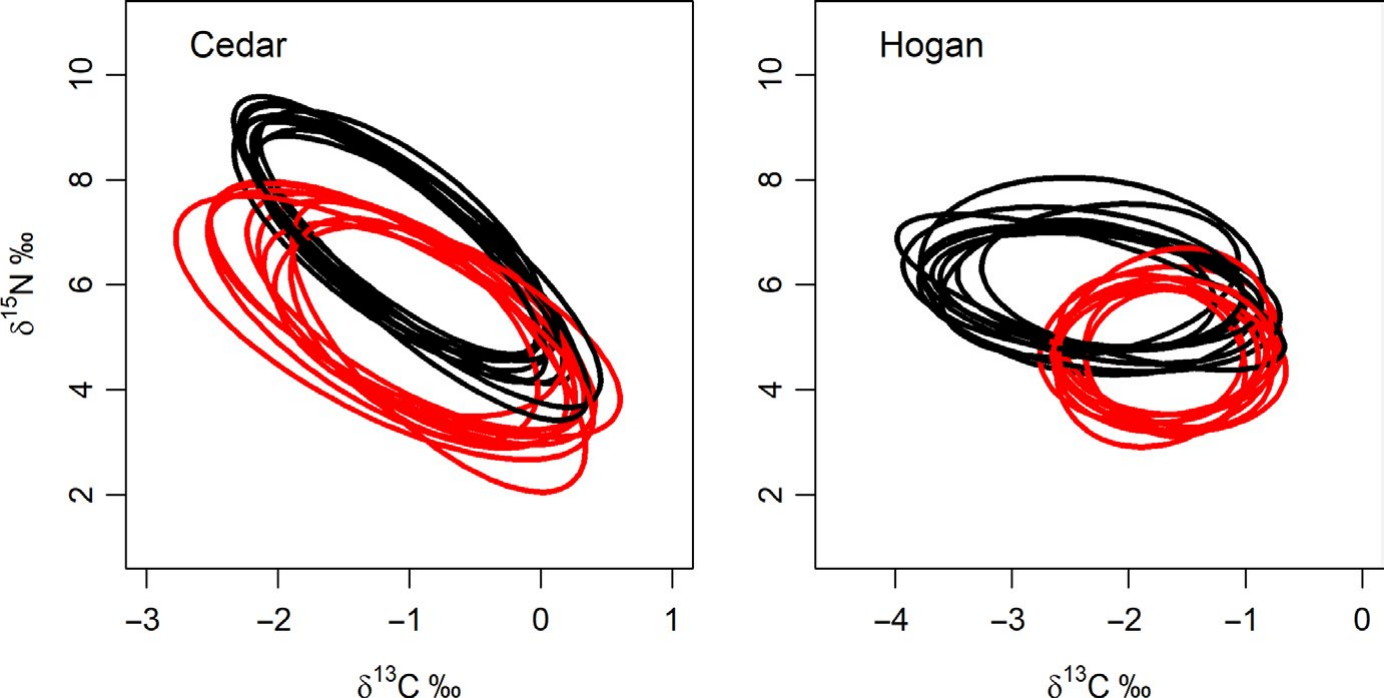
Plausible isotopic niche space for blackfin and cisco forms in Cedar and Hogan Lakes based on bimodality in gill raker number (Bell et al., 2019). Axis values are δ^15^N and δ^13^C relative to baseline values for each lake (δ^15^N_fish_ − δ^15^N_base_; δ^13^C_fish_ − δ^13^C_base_). Note the scale of relative δ^13^C varies with each figure panel

Based on this result, ciscoes captured in Cedar and Hogan Lakes (blackfin and cisco) were compared based on gill raker groupings (Figure 5; relatively low gill raker count for cisco and relatively high gill raker count for blackfin; Bell et al., 2019). The isotopic niche size of blackfin and cisco was similar in size in Cedar Lake (Figure 5; blackfin SEA_B_ = 1.01; 95% CI, 0.74–1.34; cisco SEA_B_ = 1.40; 95% CI, 0.92–2.06). The probability of Cedar Lake cisco being in the niche space of Cedar Lake blackfin was 69.5 (95% CI, 42.4–92.2) while the probability of a blackfin being in the niche space of cisco was 46.5 (95% CI, 31.4–64.9) (Figure 5). In Hogan Lake, blackfin had a larger niche space than cisco (Figure 5; blackfin SEA_B_ = 1.04; 95% CI, 0.56–1.69 vs. cisco SEA_B_ = 0.70; 95% CI, 0.5–0.95). The probability of Hogan Lake cisco being in the niche space of Hogan Lake blackfin was 32.0 (95% CI, 12.4–50.2) while the probability of a blackfin being in the niche space of cisco was 46.4 (95% CI, 26.5–75.1), a nearly identical overlap probability for Cedar Lake blackfin in cisco isotope space.

The trophic position of Cedar Lake blackfin ($\bar{x}$ TP = 3.98; *SE* = 0.046) was higher than for Cedar Lake cisco ($\bar{x}$ TP = 3.54; *SE* = 0.058) as was Hogan Lake blackfin ($\bar{x}$ TP = 3.76; *SE* = 0.44) relative to Hogan Lake cisco ($\bar{x}$ TP = 3.38; *SE* = 0.029). Blackfin trophic position in each lake was significantly higher than trophic position of co‐occurring cisco (Cedar Lake, Wilcoxon rank sum = 968, *p* < .001; Hogan Lake, Wilcoxon rank sum = 471, *p* < .001).

## 4 DISCUSSION

Isotopic niche size of cisco at least doubled in size in lakes with *Mysis* relative to lakes with *Chaoborus*, the two dominant diel migrators in the lake set. Use of different habitats (benthic and pelagic) and the presence of different forms (cisco and blackfin) further increased isotopic niche size for cisco in *Mysis*‐based food webs. These results support the hypothesis that *Mysis* presents ecological opportunity for the ancestral form of cisco leading to increased use of different habitats in some lakes, and arguably phenotypic diversity as well (Eshenroder et al., 1998; Eshenroder & Burnham‐Curtis, 1999). Blackfin in Algonquin Park prey on *Mysis* (Bell et al., 2019), and we found blackfin to have a higher trophic position relative to co‐existing cisco indicating that *Mysis* have inserted a trophic position step relative to pelagic cisco in the same lake (Vander Zanden et al., 1999). The results of this study have three important implications for understanding cisco niche diversity that for decades proved challenging because of the range of forms, complex glacial history, and taxonomic confusion.

First, niches of coregonine fishes following ecological speciation, including isotopic niche, are relatively conservative and repeatable (Harrod et al., 2010; Siwertsson et al., 2013). We found repeatable patterns of relative overlap of isotopic niches between cisco and blackfin in Cedar and Hogan Lakes, two ecosystems functionally separate for the past 12,000 cal yrs BP (Table 6; Figure 5). Furthermore, the benthic blackfin had a larger SEA_B_ than co‐occurring cisco, with pelagic cisco greatly overlapping with blackfin (81% and 97% overlap for Cedar and Hogan Lks, respectively; Table 6). Blackfin overlapped much less with cisco in their isotopic niche (49% and 41% for Cedar and Hogan Lakes, respectively) pointing to blackfin as occupying different and wider isotopic niche space relative to cisco. For cisco in relatively small inland lakes, ecological speciation has repeatedly resulted in pelagic cisco, typical of the species (ancestral niche) and likely the founding form (Piette‐Lauzière et al., 2019; Turgeon et al., 2016), co‐occurring with a single benthic form of different morphology, gill raker counts and coloration in some cases (Bell et al., 2019; Piette‐Lauzière et al., 2019; Turgeon et al., 2016). Weight of evidence points to ecological speciation in each case as the basis of adaptive radiation. Here the ancestral form, cisco, retains smaller pelagic isotopic niches and a relatively narrow morphological space (Figure 4, PCA ordination) while the derived form, blackfin, expanded its isotopic niche toward exploitation of *Mysis*, and perhaps including other planktonic or benthic prey as well.

Niche conservatism has been detected in lakes where loss or recovery of coregonine forms has occurred. In the Laurentian Great Lakes (Blanke et al., 2018) and Lake Constance (Vonlanthan et al., 2012), loss of phenotypic diversity with the disappearance of recognizable forms resulted in contraction of overall isotopic niche space among remaining forms rather than expansion among surviving forms to occupy the vacant isotopic niche. In Lake Constance, coregonine phenotypic diversity and isotopic niche have recovered under improving water quality conditions with the re‐occurrence of a form once thought lost in the lake (Jacobs et al., 2019; Vonlanthen et al., 2012). These outcomes point to relative niche conservatism among coregonines as a repeatable element of the phenotype–environment correlation (Harrod et al., 2010), and as found with *Anolis* lizards (Gavrilets & Losos, 2009; Losos, 2010), cisco diversity is repeatable among lakes under similar environmental conditions.

Second, cisco diversity is associated with *Mysis*‐based lake food webs. To date, descriptions of cisco diversity, including based on gill raker counts (e.g., this study; Eshenroder et al., 2016; Etnier & Skelton, 2003; Turgeon & Bernatchez, 2003; Turgeon et al., 2016), have been from lakes with *Mysis* as a member of the zooplankton assemblage. In some cases, descriptions of *Mysis* in the diet of some cisco forms are part of the diversity assessment. Cisco isotopic niche size in the two lakes with *Chaoborus* as the dominant diel migrator was less than 50% of the isotopic niche size of monomorphic cisco in two lakes with *Mysis*. We found cisco diversity, both behavioral and phenotypic, to be associated with *Mysis* lakes in our study region of Algonquin Park. This includes two lakes (Three Mile and Cauchon) where cisco without any clear morphological differences were foraging in different habitats (Figures 2 and 3). This agrees with the proposition that behavioral changes, detected here as foraging habitat, precedes morphological divergence (Piette‐Lauzière et al., 2019). *Mysis* expands isotopic niche, but not always in the same morphological direction. In White Partridge Lake of Algonquin Park, Turgeon et al. (2016) found another pair of cisco forms that co‐occur with *Mysis*. In this lake, the benthic form appears to converge in many morphological characteristics with shortjaw. However, the presence of *Mysis* does not necessarily lead to phenotypic or habitat use diversity. Two *Mysis* lakes in this study (Grand and Manitou Lakes; Figure 2) had only pelagic foraging cisco with a phenotype typical of the species. *Mysis* is therefore sufficient to expand isotopic niche size of cisco, as we found, but not sufficient in all cases to yield an expansion of behavioral or morphological diversity.

Third, the depth gradient of lakes is the opportunity axis for radiation of cisco diversity. Adaptive radiation in cisco is associated with occupancy and evolution of forms in deeper lake habitats than occupied by the ancestral pelagic form. Virtually all native cisco diversity in the Laurentian Great Lakes is collectively referred to as the deep‐water ciscoes. In this study, blackfin or cisco captured foraging on lake bottom (Cedar, Hogan, and Cauchon Lakes) occupied wider ordination space on the first principle component based on size, morphology, gill rakers, and isotopic niche data relative to pelagic cisco (Figure 4). Because pelagic cisco is regarded as the ancestral form, this result points to increasing diversity on a depth gradient as described by Eshenroder and Burnham‐Curtis (1999) for the Laurentian Great Lakes. Consuming *Mysis* is associated with an increase in trophic position of benthic‐captured blackfin in Algonquin Park lakes relative to cisco co‐occurring in Cedar and Hogan Lakes.

Hogan Lake SEA_B_ was lower than Cedar and Radiant Lakes and appeared to be a departure from the pattern of increasing isotopic niche size corresponding to phenotypic diversity (Figure 2). One possible explanation may be the strongly asymmetric basin of Hogan Lake where one smaller region of the lake has sufficient bathymetric depth to retain a full cold‐water community (including ciscoes, lake whitefish and lake trout) with the dominant predator, lake trout, confined to this basin during periods of lake stratification (Dolson et al., 2009; McCann, 2012). For parts of the annual temperature cycle, cold‐water fish are excluded from large areas of asymmetric lakes. The annual sequence of seasonal confinement to deeper but limited cold‐water habitat in summer, followed by release in winter and access to previously restricted foraging habitat, may generate spatial structure for cisco and blackfin on a seasonal basis that differs from single basin lakes. For an isotopic niche, this may result from seasonal contrasts in prey type, such as summer that includes *Mysis*, versus winter that allows for foraging in large shallow areas of asymmetric lakes without *Mysis* and alternative prey including benthic prey.

Cisco in Cauchon Lake forage in the pelagic zone and in benthic habitat. The PCA analysis indicated some differentiation between the two foraging behaviors (Figure 4) and relatively little overlap in isotopic niche size (Table 6; Figure 3). There was insufficient phenotypic or genetic diversity to conclude two forms are present as occurred in Cedar and Hogan Lakes (Piette‐Lauzière et al., 2019). As well, gill raker counts are unimodal and consistent with *C. artedi* (Bell et al., 2019). Cauchon Lake is relatively deep for its surface area (mean depth = 13.5 m; max. depth = 43.0 m; surface area = 232.2 ha) so pelagic and benthic habitats may represent alternative foraging niches utilized by cisco. The behavioral separation of cisco in Cauchon Lake may represent a stage of partial separation of forms but incomplete ecological speciation (figure 4 in Piette‐Lauzière et al., 2019). Given these similarities among cisco in Cauchon Lake then small separation of forms detected in ordination space suggest an effect of the δ^15^N aspect in the isotopic niche in this lake. Dichotomous habitat use in Cauchon Lake suggests that *Mysis* movement and distribution may support alternative foraging niches for cisco given its relative depth to surface area but not enough spatial separation of foraging niches to support phenotypic diversification. Whether ciscoes are monomorphic, demonstrate expanded use of alternative foraging habitats or the evolution of distinct forms, *Mysis*‐based lake food webs provide ecological opportunity for cisco diversification in some lakes but not all lake food webs.

*Mysis* movement and habitat occupancy in lakes are complex and related to depth. Large adult stages may remain on lake bottom while smaller stages utilize diel vertical migration for foraging. The relative representation of this pattern appears to be a function of depth (O'Malley et al., 2018). O'Malley et al. (2018) found similar abundances of *Mysis* in benthic habitat day and night and in pelagic habitat day and night at 100 m in Lake Champlain but not at 60 m where diel movements were common among smaller *Mysis*. In Lake Superior, similar patterns of *Mysis* distribution have been detected with smaller stages migrating vertically at night and larger stages remaining closer to bottom (Bowers, 1988). The complex movement and habitat occupancy by *Mysis* as a function of depth suggest multiple fitness peaks based on a trade‐off minimizing vulnerability to predation and maximizing growth potential at different depth zones. If the pattern of *Mysis* distribution detected in Lake Champlain is a general occurrence, then depths of 100 m or greater may provide fitness peaks in the cisco/ *Mysis* predator prey system that can support cisco diversification as a function of depth—an expansion of the earlier hypothesis of cisco buoyancy evolution to exploit *Mysis* at depth (Eshenroder et al., 1998; Eshenroder & Burnham‐Curtis, 1999).

Multiple fitness peaks for *Mysis* as a function of depth also suggest multiple fitness peaks for cisco both as predators in pursuit of *Mysis* as prey and as prey evading predation from species such as lake trout. In this study, lakes were all less than 100 m in depth yet several possessed cisco diversification represented as expanded habitat use or phenotypic diversification stemming from ecological speciation (Bell et al., 2019; Piette‐Lauzière et al., 2019). Other lakes in this study with *Mysis* did not show any diversification in habitat use or phenotype. The presence or absence of cisco diversity may reflect differences in *Mysis* density among lakes and/or whether multiple fitness peaks persist for *Mysis*, perhaps related to basin morphology and depth. The density of predatory fish and their ability to detect prey including cisco at depth are also likely contributing to cisco habitat utilization as shown in other studies (e.g., Ahrenstorff et al., 2011; Hrabik et al., 2006; Jensen et al., 2006). This study did not incorporate enough lakes to quantitatively assess the strength of these factors in driving cisco diversity. In inland lakes with *Mysis*, cisco forms may evolve as purely benthic foragers (Turgeon et al., 2016) or as blackfin as we found in some Algonquin Park lakes. Differences among lakes in cisco forms may reflect differences in *Mysis* habitat residency or migratory behavior and corresponding ecological opportunities for cisco diversification.

Cisco diel movements can be complex in lakes with or without *Mysis*. Cisco partial daily vertical migration, no migration, and other types of behavior have been found in lakes with *Chaoborus* or other planktonic assemblages but without *Mysis* (Ahrenstorff et al., 2013). We did not find any indication of expanded habitat use by cisco in the two *Chaoborus* lakes in this study. *Chaoborus* and other planktonic assemblages may not provide consistent fitness peaks as a function of depth or prey profitability that is potentially present in *Mysis*‐based food webs. Given the capacity of *Mysis* to act as a food web engineer (Vander Zanden et al., 1999), the resulting planktonic composition and size structure may provide an adaptive trophic landscape that persists through time (or minimizes variability) that in turn provides ecological opportunity for cisco. This may also define the replicated diversity noted for cisco across inland and large lake ecosystems with *Mysis* (Piette‐Lauzière et al., 2019; Turgeon & Bernatchez, 2003; Turgeon et al., 1999, 2016), and not colonization via glacial lake flooding that has historically been invoked, including the assumption of shared phylogenetic history, to account for consistency in cisco diversity across landscapes (Clarke, 1973; Dymond & Pritchard, 1930; Etnier & Skelton, 2003; Smith & Todd, 2004).

In conclusion, ecological opportunity is central in adaptive radiations including expanded habitat use and eventual clade diversification (Stroud & Losos, 2016). This pattern has been shown repeatedly in many cases, including *Anolis* lizards (Mahler et al., 2010; Martin & Wainwright, 2013), several fish species (Schluter, 1996; Schluter & McPhail, 1992), and other organismal groups. We found *Mysis* expands cisco isotopic niche size relative to cisco in *Chaoborus*‐based food webs and significantly so in 6 of 7 *Mysis* lakes. Isotopic niche size continued to increase for cisco with expanded habitat use and phenotypic diversity. This pattern of expanded trophic niche is a necessary condition for the *Mysis* hypothesis to account for not only diversity in the Laurentian Great Lakes but in other lake ecosystems as well.

## CONFLICT OF INTEREST

None declared.

## AUTHOR CONTRIBUTIONS

**Mark S. Ridgway:** Conceptualization (lead); formal analysis (equal); funding acquisition (lead); investigation (equal); methodology (equal); project administration (equal); supervision (lead); writing – original draft (lead); writing – review and editing (lead). **Gabriel Piette‐Lauzière:** Conceptualization (equal); data curation (equal); formal analysis (equal); validation (equal); visualization (equal); writing – review and editing (supporting). **Allan H. Bell:** Data curation (lead); formal analysis (equal); investigation (equal); software (equal); validation (equal); visualization (equal); writing – review and editing (supporting). **Julie Turgeon:** Conceptualization (supporting); funding acquisition (lead); project administration (equal); supervision (equal); writing – original draft (supporting); writing – review and editing (equal).

## Data Availability

Sample isotopic data, PCA input data and R code are available in Dryad: https://doi.org/10.5061/dryad.5hqbzkh42.
